# A Smart Spoofing Face Detector by Display Features Analysis

**DOI:** 10.3390/s16071136

**Published:** 2016-07-21

**Authors:** ChinLun Lai, ChiuYuan Tai

**Affiliations:** Department of Communication Engineering, Oriental Institute of Technology, New Taipei City 220, Taiwan; layak.day@broadtec.com.tw

**Keywords:** spoofing action detector, non-intrusive anti-spoofing face liveness detection, probabilistic neural network, biometric authentication system cheat, display features analysis

## Abstract

In this paper, a smart face liveness detector is proposed to prevent the biometric system from being “deceived” by the video or picture of a valid user that the counterfeiter took with a high definition handheld device (e.g., iPad with retina display). By analyzing the characteristics of the display platform and using an expert decision-making core, we can effectively detect whether a spoofing action comes from a fake face displayed in the high definition display by verifying the chromaticity regions in the captured face. That is, a live or spoof face can be distinguished precisely by the designed optical image sensor. To sum up, by the proposed method/system, a normal optical image sensor can be upgraded to a powerful version to detect the spoofing actions. The experimental results prove that the proposed detection system can achieve very high detection rate compared to the existing methods and thus be practical to implement directly in the authentication systems.

## 1. Introduction

Biometrics technology is a unique approach for recognizing human features/behaviors based on physical and chemical properties. The most frequently seen approaches are based on fingerprints, human face, iris, hand geometry, dorsal vein, signature, voice, and DNA. In recent years, due to the multiple convenient qualities (quick, remote detection ability, non-contact), face detection has been widely applied to access control, monitoring and focusing systems for the verification of the subject’s identity and behavior. With the widespread adoption of biometric technology, the techniques of spoofing have become increasingly advanced with biometric information being forged or collected to deceive or bypass the verification of a biometric system [1]. It is thus crucial for the biometric system to identify the forged characteristics.

**Figure 1:**
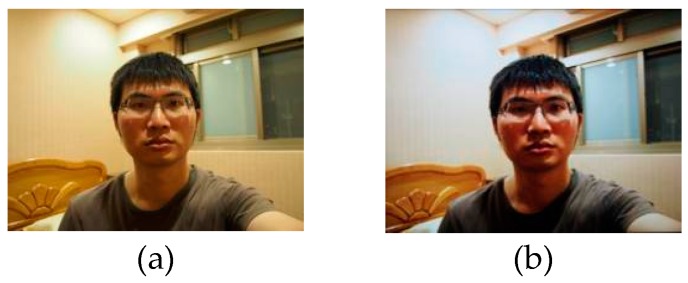
Spoofing the biometric system with retina identification technology. Demonstration of spoofing the biometric system with retina resolution display, (**a**) the actual filmed image, and (**b**) image reconstruction by an iPad with retinal display.

Regarding human face detection technology, it is rather easy for the imposter to collect forged data by using social networks or digital camera. A valid identity can be fabricated by using any of the following three methods: (1) having a photo of a valid user; (2) having a video of a valid user; or (3) having a 3D facial model or mask. For example, Figure 1 demonstrates a normal spoofing case where a face recognition system is cheated and accessed by the spoofed face image, displayed in a high resolution retina display, rather than an actual filmed face image.

The face spoofing detection technology has developed fast in recent years [2,3,4]. Some of the used methods include dynamic detection [5,6], static detection, spatial frequency or time frequency [7,8], and two dimensional or three dimensional characteristics classifications [9]. For example, taking the display monitor feature into consideration, Peixoto et al. [10] and the extended technologies [11] show that the brightness of the LCD screen will cause the edge of the images to become blurry. A recessive reflection coefficient characteristic has been raised and the image analysis using histogram equalization has been included. Using the human face database from NUAA and Yale to test the result, it was revealed that such characteristics classification reduced 50% of detection errors based on high definition photos from the NUAA database. For the Yale database, the successful rate of face spoofing detection using LCD screen was approximately 65%.

As stated in the article by Allan da Silva Pinto [12], a visual ridge frequency analysis based on the Fourier spectrum analysis was established to determine if the image comes from the LED or LCD screens. On the other hand, Jiangwei Li [13] used Fourier spectrum analysis to detect the changes in the facial movement sequences. Hyung-Keun Jee [14] used the Hamming distance to measure the movement of the eyes to verify a live face. W. Bao, and H. Li, et al. [15] verified the differences between the three dimensional human faces and the two dimensional images based on the different optical flow. W. R. Schwartz [16] utilized the spatial and time messages of the low-level feature descriptors to differentiate between the authentic faces and the spoofed faces, while J. W. Li [17] used multiple Gabor responses to detect the blinking of the eyes and verified the differences between authentic human faces and the spoofed faces in the two dimensional images. Moreover, Chin-Lun Lai [18] used an intuitive concept to detect the fake face when sufficient display borderlines are found.

The differences of the methods mentioned above are the efficiency of the processing and the success rate of the detection. In this paper, a novel face spoofing solution is proposed to prevent the biometric system from being “deceived” by the video or picture of a valid user that the counterfeiter took with a high definition handheld device (e.g., iPad with retina display). To efficiently and accurately detect the spoofed faces, a method that can identify the forged faces rapidly based on the information of a single image is adopted. Since most of the high definition display monitors use an LED as backlight module, it is observed that LED emits light by first exciting the phosphor with high-power short wave blue light and the low-power yellow light is then generated and converts a portion of the blue light into white light. Based on this premise, it is possible to detect the display monitor by verifying the chromaticity regions on the image and establishing an expert decision-making model with a probabilistic neural network (PNN) approach. Thus, face spoofing detection can be achieved as well. By analyzing the characteristics of the display monitor and the learning ability of the neural network and adopting the tandem identification technique, the successful rate of face spoofing detection can exceed 95% in a single shot image, which has an advantage over the previous ones. Thus, the reliability of the corresponding biometric identification system will be greatly improved.

This paper is organized as follows: Section 2 describes the design concept and principal theory of the proposed spoofing detection method, while the designed algorithm is described in Section 3. Section 4 states the experimental methods and the test results as well as the discussions. Finally, the conclusion and future work are presented in the last part.

## 2. Design Concept and Principal Theory

### 2.1. Features of Current LED-Backlight Display

To understand the design concept of the proposed method, some basic principles about LED/LCD display should be revealed first. The light emitted from a light-emitting diode (LED) has a specific wavelength and thus a specific color. The latter depends on the LED’s semiconductor material. LED semiconductors consist of combinations of elements such as phosphides or arsenides. There are various combinations, each of which releases varying amounts of energy according to the semiconductor material’s band gap. When charge carriers are recombined, photons are emitted according to specific discrete energy levels. This specifies the particular light color. For example, blue light is produced if a high level of energy is released and red light is produced if a lower level of energy is emitted. Thus, monochromatic (single color) light is produced. The following is LEDs special feature: Each LED light color is limited to a very narrow range of wavelength (keyword: dominant wavelength) which accordingly only represents a specific light color. The only spectrum that cannot be produced directly from the chip is the white light spectrum, since white light represents a mixture of all light colors.

**Figure 2:**
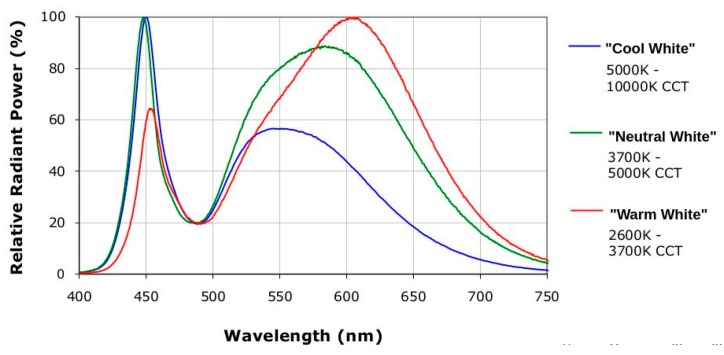
An example spectrum of most of the white color light-emitting diodes (LEDs).

The current procedure for producing white light is the principle of photoluminescence. A thin phosphorus layer is applied on top of a blue LED. The LED’s shortwave energy-rich blue light, as illustrated in Figure 2, stimulates the phosphorus layer to light up and it emits lower-energy yellow light. Part of the blue light is thus transformed into white light. The white light’s color tone can vary with the metering of the phosphorus colorant. Different white tones, such as warm white, neutral white or cold white are thus produced.

### 2.2. Color Space Analysis for LED Displays

**Figure 3:**
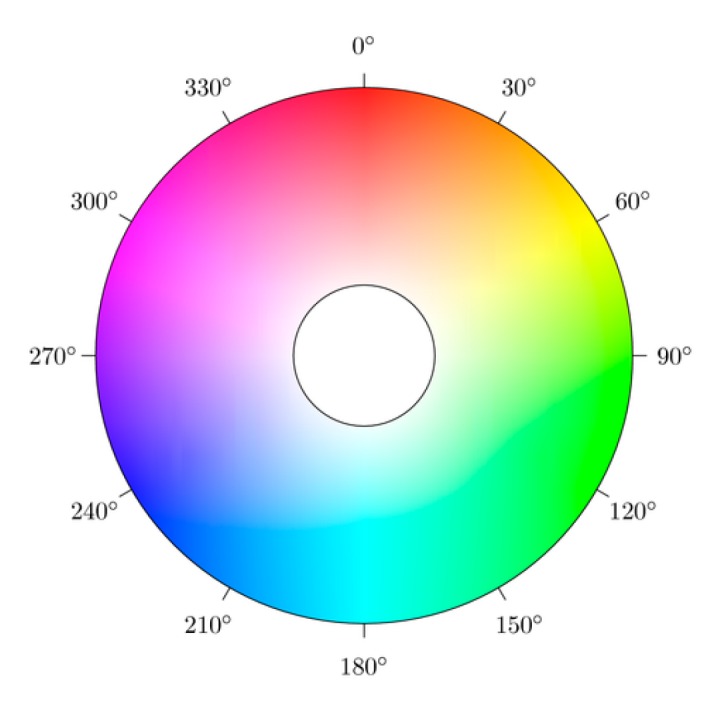
The color space of hue vector.

HSV is a cylindrical-coordinate representation of points in an RGB color model. HSV refers to Hue, Saturation and Value. The conversion process of RGB to HSV is as follows:

Let (r,g,b) be the red, green and blue coordinates of a certain color with their values being real numbers between zero and one. Set “max” as the r, g or b coordinate with the maximum value and “min” as the minimum value. The value of h has been normalized between $0^{{}^{\circ}}$ to $360^{{}^{\circ}}$ which can be obtained by

(1) $$h=\left\{\begin{array}{c} 0^{{}^{\circ}},\;\mathrm{if}\;\max=\min\\ 60^{{}^{\circ}}\times \frac{g-b}{\max-\min}+0^{{}^{\circ}},\;\mathrm{if}\;\max=r\;\mathrm{and}\;g\geq b\\ 60^{{}^{\circ}}\times \frac{g-b}{\max-\min}+360^{{}^{\circ}},\;\mathrm{if}\;\max=r\;\mathrm{and}\;g<b\\ 60^{{}^{\circ}}\times \frac{b-r}{\max-\min}+120^{{}^{\circ}},\;\mathrm{if}\;\max=g\\ 60^{{}^{\circ}}\times \frac{r-g}{\max-\min}+240^{{}^{\circ}},\;\mathrm{if}\;\max=b \end{array}\right\}$$

and the color space of the hue is shown in Figure 3.

**Figure 4:**
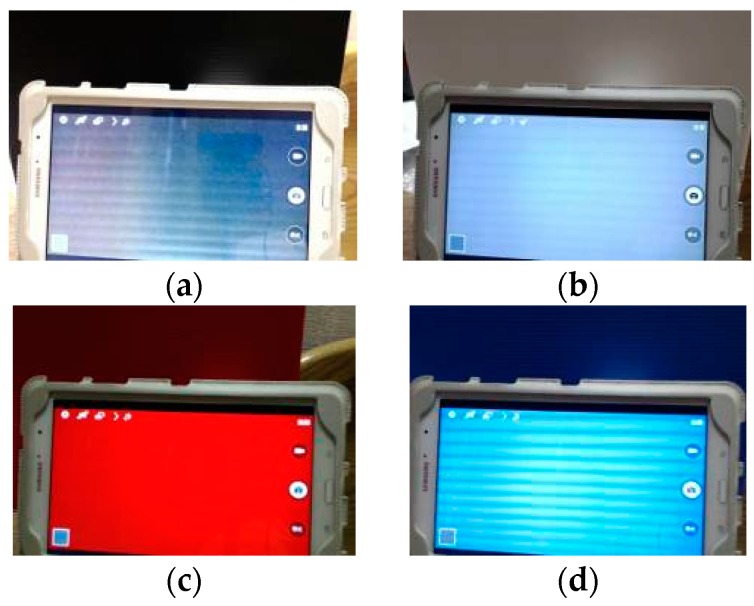
The four kinds of original color image. Original image of the displayed color for (**a**) black; (**b**) white; (**c**) red; and (**d**) blue.

**Figure 5:**
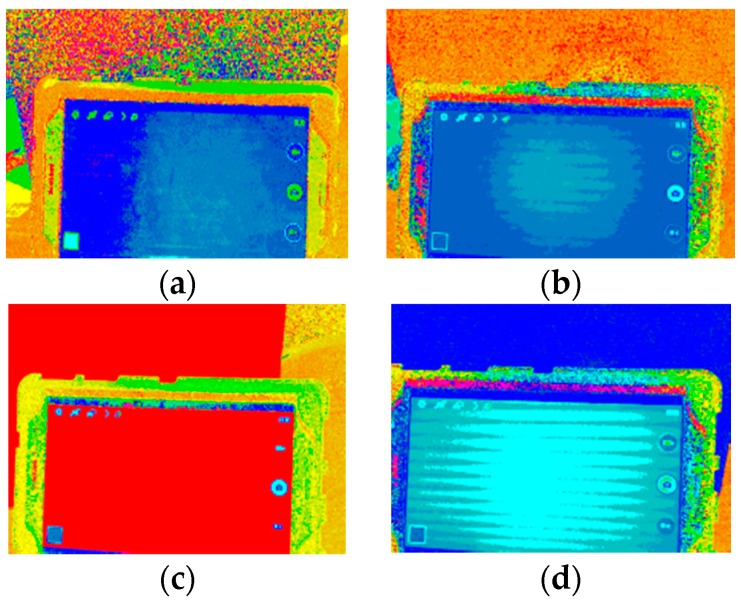
Comparison images of the corresponding colors shown on the high definition LED display monitor. The LED compared images for (**a**) black; (**b**) white; (**c**) red; and (**d**) blue.

To find the implicit difference between the natural image and LED displayed image, all types of colors have been presented on the high definition display monitors and compared with the color swatches to see the hue changes. As shown in Figure 4 and Figure 5, the captured images have been processed. Both saturation and value have been set at 1 to eliminate their influences. After presenting the outcome in RGB, it was revealed that both black and white colors appeared to have a blue hue on the high definition display monitors.

Based on the experimental result, it is assumed that white light is generated due to the stimulation of blue light LED and both black and white colors are presented by white light. As a result, both colors tend to be bluish. These observed results, however, offer an explicit cue for detecting a LED monitor in the captured image, and thus provide us with implicit evidence of fake faces. That is, observing the dark and bright regions of a face image, it can be concluded that a fake face is detected if a high ratio of blue color region is present.

## 3. The Designed Algorithms

In this section, the fake face detection procedure is proposed, the corresponding function blocks include face features positioning, color space transform and analysis, and expert decision model by PNN structure, and these algorithms are described in the following subsections.

### 3.1. Face Features Positioning and Preprocessing

**Figure 6:**
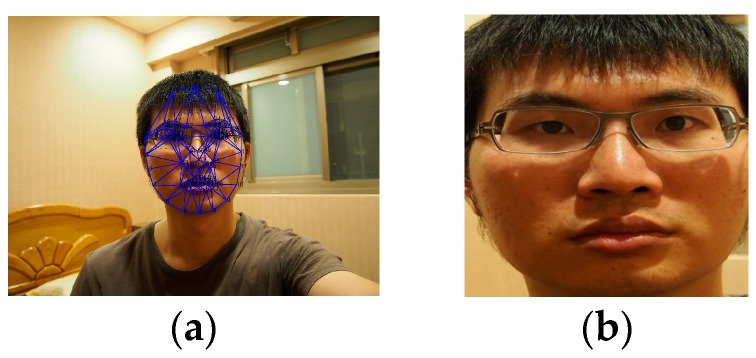
Face image with the region of interest (ROI) being identified and captured by the STASM algorithm. (**a**) Capturing the region of the face with STASM; and (**b**) Converting the image into the resolution of 320 × 320.

**Figure 7:**
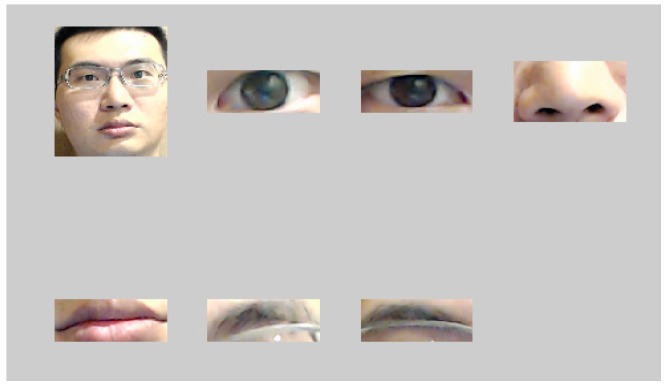
The interest face features of eyes, nose, mouths, and eyebrows.

First of all, the face is targeted using normal face detection algorithms such as AdaBoost filter. Once the face is found, a total of 68 characteristic points are positioned on each of the subjects’ faces by adopting active shape model (ASM) technology [19]. One of the famous algorithm to find the characteristic points are STASM which is a C++ software library. As shown in Figure 6a, these characteristics helped us to capture the region of interest (ROI), direction and the position of the face. To unify the subsequent analyses, the captured face image was normalized into the resolution of 320 × 320 as shown in Figure 6b. After examining a number of face images, it is found that the colors white and black tend to appear in eyebrows, eyes, nose and mouth (as shown in Figure 7). These face parts were thus selected as identifiable characteristics.

### 3.2. Color Space Transform and Analysis over ROI

**Figure 8:**
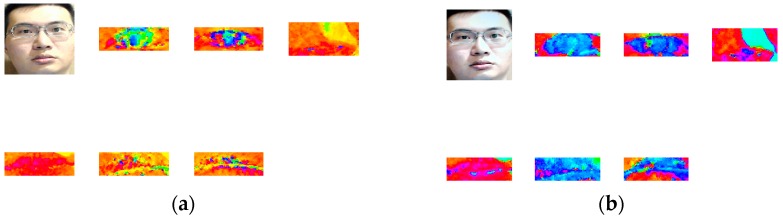
Hue, Saturation and Value (HSV) images of the authentic image and the spoofed image. The eyes, nose, mouse, and eyebrows feature images in HSV space. (**a**) The original (authentic) face; (**b**) The reproduced (spoofed) face.

Once the interest face regions (eyes, nose, mouse, and eyebrows) are segmented, the HSV color space transformation is conducted on these ROI image parts, as shown in Figure 8. By comparing the original and reproduced (by LED display) images, it is found that there exists a big difference in the hue distributions of the authentic image and the spoofed image. This is described as follows.

**Figure 9:**
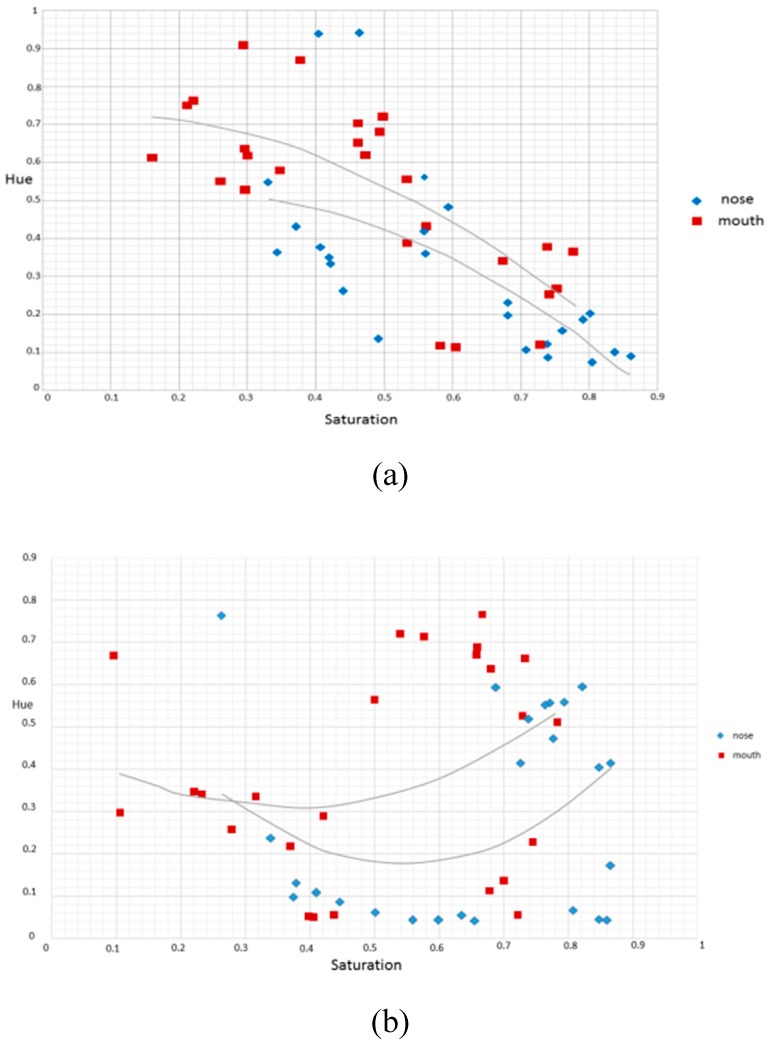
The relation between the saturation and the average hue of the authentic image (left) and the spoofed image (right) using nose (blue) and mouth (red) as examples. (**a**) real image; and (**b**) spoofed image.

After examining the subject’s nose and mouth, it is revealed that both parts tend to be reddish in terms of the average hue. As shown in Figure 3 of hues diagram, the distribution of the color blue was from 0.75 to 0.5 while the color red was from 0 to 0.18 and 0.825 to 1. It was also discovered from Figure 8 that most of the authentic images’ average hues fell within the red region while the spoofed images’ average hues fell within the blue region during the high saturation state. As the saturation decreased, the average hues of the authentic images moved toward the blue region while that of the spoofed images moved toward the red region. The phenomenon presented in Figure 4 suggested that, as the saturation increases, blue LED—in an attempt to excite more white lights—enhances accordingly. If the saturation decreases, the blue light weakened gradually. The result is as shown in Figure 9.

**Figure 10:**
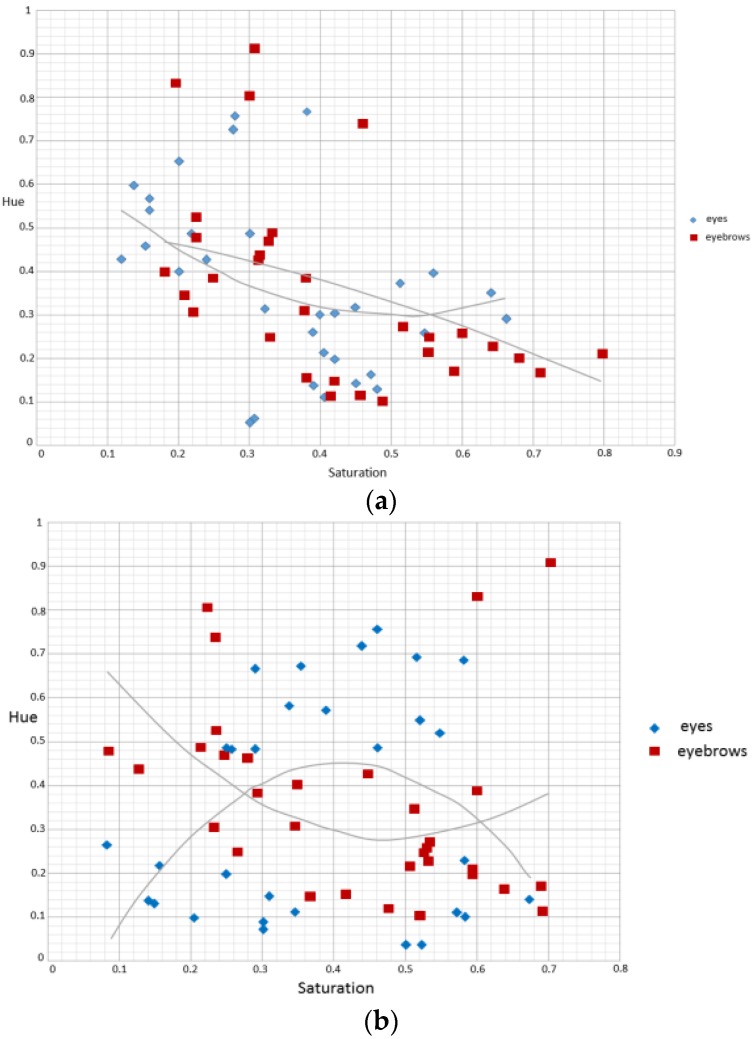
The relation between the saturation and the average hue of the authentic image (left) and the spoofed image (right) using eyes (blue) and eyebrows (red) as examples. (**a**) authentic image; and (**b**) spoofed image.

On the other hand, in terms of the eyes and the eyebrows, it was discovered (from the Figure 10) that almost all of the authentic images fell outside of the blue region. Affected by the glasses, some authentic images of the eyes fell within the blue region. The spoofed images tended to gather around the blue region during medium saturation state.

**Figure 11:**
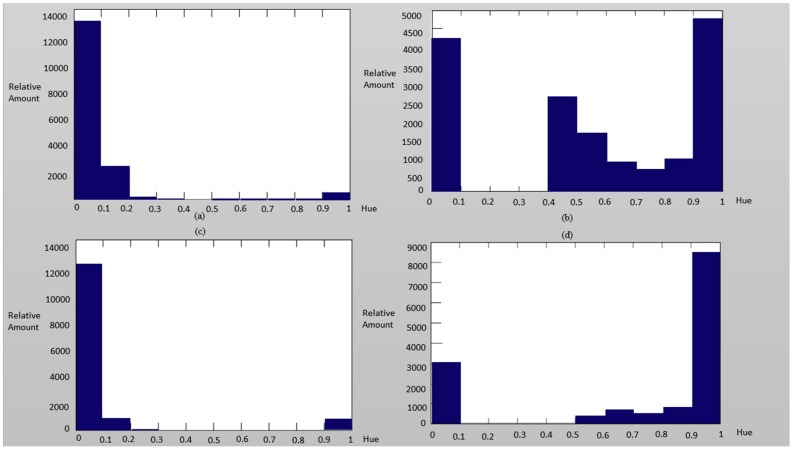
The hue distribution of the authentic image of (**a**) nose, (**c**) mouth,) and the spoofed image of (**b**) nose, (**d**) mouth as examples.

**Figure 12:**
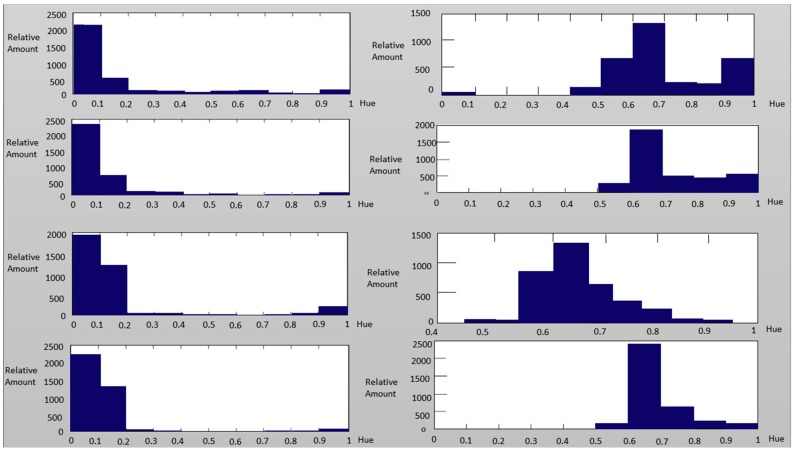
The hue distribution of the authentic image (Left column) and the spoofed image (Right column) using eyes (Upper two rows) and eyebrows (Lower two rows) as examples.

The above description about the hue distributions for real and fake faces can also be approved by observing Figure 11 and Figure 12. That is, the hue of the spoofed images tended to reach the blue region.

### 3.3. Expert Decision Making by PNN Model

To identify the aforementioned complex characteristics information, the result analysis was adopted to establish an expert decision-making model with probabilistic neural network (PNN) being used as a simulation tool. PNN is a supervised network architecture proposed by D. E. Specht [20] which can rapidly learn from a set of training data. With enough training data at hand, it had been proved that PNN converges asymptotically to the Bayesian classifier. The most important task within the Bayes classification rule is to estimate the probability density function (PDF)—$f_{A}(x)$—of each class A from a set of data.

(2) $$f_{A}(x)=P(x|A)$$

where x is the input data to be classified. Parzen [21] has proved that any smooth and continuous PDF can be asymptotically approached by a set of predictors. On the other hand, Specht in 1990 proposed a special estimate function for Equation (2) as follows:

(3) $$f_{A}(x)=\frac{1}{{\left(2\pi \right)}^{p/2}\sigma^{p}}\frac{1}{n_{t}}\sum_{i=1}^{n_{t}}\exp[\frac{-{\left(x-x_{A_{i}}\right)}^{t}(x-x_{A_{i}})}{2\sigma^{2}}]$$

where p is the dimension of input data, $n_{t}$ is the number of training data, $x_{A_{i}}$ it the i-th training data in class A, while σ denotes the smoothing parameter.

It is observed from Equation (3) that $f_{A}(x)$ is the sum of $n_{t}$ multivariate Gaussian distributions and its center points are each of the training data. The sum is not restricted to be Gaussian function. This predictor applies to the general classification questions. Therefore, Specht proposed the PNN architecture to implement the estimation of $f_{A}(x)$. Within the PNN, the training data and the data to be classified are often normalized into the vectors of the unit length. Thus, we have

(4) $${\left(x-x_{A_{i}}\right)}^{t}(x-x_{A_{i}})=-2(x^{t}x_{A_{i}}-1)$$

After that, Equation (4) can be simplified as the form

(5) $$f_{A}(x)=\frac{1}{{\left(2\pi \right)}^{p/2}\sigma^{p}}\frac{1}{n_{t}}\sum_{i=1}^{n_{t}}\exp[\frac{\left(x^{t}x_{A_{i}}-1\right)}{2\sigma^{2}}]$$

**Figure 13:**
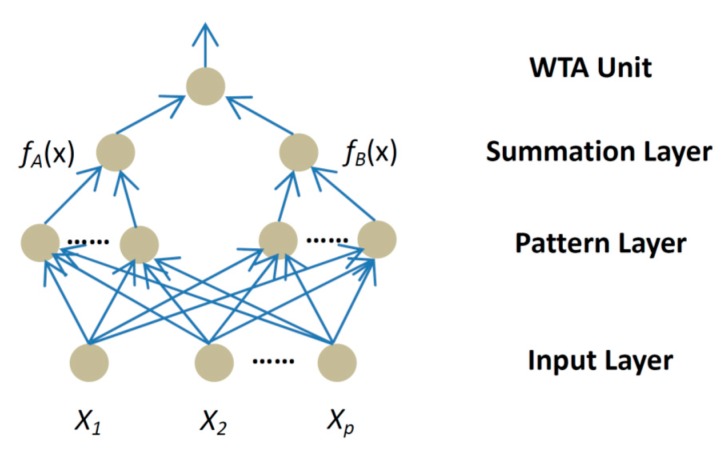
The proposed probabilistic neural network (PNN) structure.

PNN is a three layered feed forward neural network (as shown in Figure 13). The first layer is the input layer that receives the input data. The hidden layer in the middle is the pattern layer which stores all the training data. Every neuron of the summation layer corresponds to each possible class. The neuron is actually the $f_{A}(x)$ and the Equation (5) is implemented by the summation layer. If and only if the training data i belongs to class j, a connection between the pattern layer neuron i and the summation layer j exists. Within the network training stage, the training data are transferred to the pattern layer separately. The input data x to be classified is then being classified as the class with the maximum summation value $f_{A}(x)$. This is the output of the WTA (Winner-Take-All) neuron.

After completing the PNN training, the accuracy of its estimation depends on the adjustment of the smoothing parameter σ. The users have to try different σ within a certain range and select the generalized accuracy that can achieve the optimal result. Specht thus proposed another adaptive method [22] which assigned a single σ to each input neuron (or input variable). Each σ has been fine-tuned during the testing stage and those with the optimal classification result will be chosen. This task can be completed by adopting genetic algorithm. Specht further discovered that the input variables with larger genetic σ value have less influence on the predictor PDF. After repeatedly adjusting each σ value with adaptive method, the variables that are less influential to the predictor PDF can be eliminated. Such a mechanism can be further applied on the selection of the features and the reduction of the dimensions of the features.

## 4. Experiment Methods, Results and Discussions

The proposed system can determine the true or false aspects of a captured face image. The algorithms developed were programmed in C and executed under the Win7 OS platform. A lot of authentic photos taken in different environments have been garnered for this experiment and the webcam was used to collect the spoofed images which are displayed on the LED displays. The experimental equipment adopted in this paper include Olympus E-PL5 16.10 megapixel digital camera, Logitech 2M pixels webcam C920, and Samsung Galaxy Tab Pro with 2560 × 1600 resolution display. The control variables used in this experiment can be summarized in Table 1, each control variable region is divided into six rectangle sub-regions which are then used as the PNN input vectors. Therefore, 72 feature vectors are fed into the input layer of PNN.

**Figure 14:**
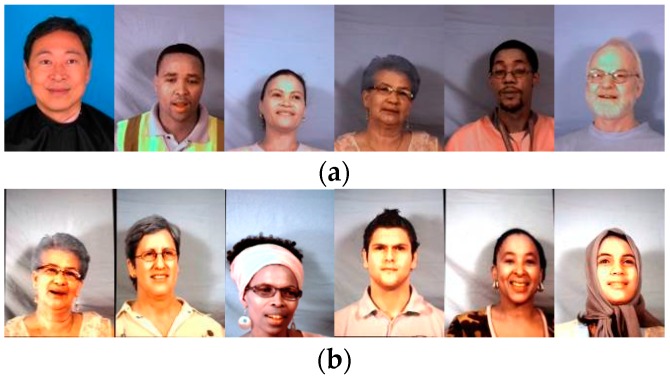
Samples of test face images (in 4557 images). (**a**) Samples of authentic images; (**b**) Samples of spoofed images (displayed by iPad).

The training of PNN applied in this paper adopted the PNN classification simulation of Matlab2014a. A total of 2277 true human face samples from **MUCT** database and the corresponding generated 3265 fake faces, some of which are shown in Figure 14, across all races, facial directions and chrominance have been classified into different categories including the authentic images without glasses, authentic images with glasses, spoofed images without glasses and spoofed images without glasses. These face samples are further divided into two sets: the training set and the testing set. The training set includes 500 real and 485 fake face samples, while the testing set includes 1777 real and 2780 fake face samples. The training set data are used as the input of PNN to learn the hidden I/O relationship. The NEWPNN module was then employed to simulate the neural network. To improve the training performance, those training vectors corresponding to the wrong detection regions detected by the STASM function are removed from the training set.

**Figure 15:**
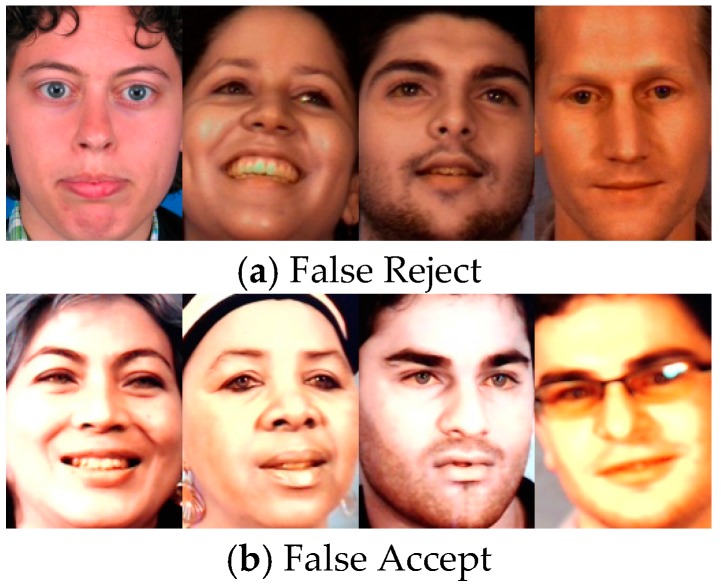
Samples of detection error cases. (**a**) False reject case images; and (**b**) False reject case images. It is observed that face samples with blue eyes more often resulted in false reject error.

After calculating all 4557 testing samples inversely, a total of 3496 samples were identified correctly and the other 1061 were misidentified. To discuss the results in more detail, 628 real faces within 1777 real faces are recognized as fake, while there are 433 fake faces within 2780 spoofed face images are recognized as real. The false rejection rate (FRR) of the system is 0.353 where it is observed that face samples with blue eyes more often resulted in false reject error. On the other hand, the false accept rate (FAR) is 0.156 and hence the average error $ER_{ave}$ (including FAR and FRR) is 0.23. The system error rate,ER, in spoofing detection system can be simply modified as ER≐FAR since fake faces are not allowed to undertake further ID recognition. Moreover, it is more easy to confirm a true face by existing methods such as [18,23]. That is, the reject ability of the proposed system for spoofing face images is near 84% for a single image. The described results are shown in Table 2 while some of the identification errors, including false acceptance and false rejection cases are shown in Figure 15.

To further improve the identification accuracy of the proposed system, three strategies are applied to improve the detection rate including time series analysis, high reflection regions removing, and detection separately for face region parts.

First, the time series analysis is similar to our previous work [23] which is used for reducing the interference of accident false acceptance error. For example, under the condition of $P_{c}=1-ER_{ave}$ accuracy (assume $p_{c}=85.4\%$ including FAR and FRR) on identifying a single image, it is possible to further apply the methodology on identifying a series samples (both work for still image and video clips). Normally, the samples are captured at the rate of $f_{s}$ frames per second (fps). A total of f frames (or $f/f_{s}$ seconds) were chosen for continuous sequence analysis. The captured face video is considered authentic if over x frames, where x≥f/2, are identified as real. The theoretical probability, $P_{T}$, which is defined as a face video being identified as authentic, can be described as

(6) $$P_{T}=\sum_{k=x}^{f}C_{k}^{f}{\left(P_{c}\right)}^{k}{\left(P_{e}\right)}^{f-k}$$

where $P_{e}=1-P_{c}$ is the error probability corresponds to which a real face is identified as a fake. For example, if $f=10,\;x=7,\;\mathrm{and}\;P_{c}=0.854$, then Equation (6) becomes

(7) $$\begin{array}{c} P_{T}=C_{7}^{10}{\left(0.854\right)}^{7}{\left(0.146\right)}^{3}+C_{8}^{10}{\left(0.854\right)}^{8}{\left(0.146\right)}^{2}+\\ C_{9}^{10}{\left(0.854\right)}^{9}{\left(0.146\right)}^{1}+{\left(0.854\right)}^{10}=0.9542 \end{array}$$

The correct identification rate now is much better than the previous one (0.854) where only one frame is referred. Accordingly, some practical adopted examples (where x≥f/2, $P_{c}=0.854$) are listed in Table 3.

It is observed in Table 3 that adjusting the number of frames captured and that of the frames identified as authentic can effectively increase the success rate of the video identification. That is, a correct identification rate near 99% (within 1 second period) is possible. That is, if the identification rate for a single frame is not high enough, the overall system performance can be improved to be practical by longer time period analysis. That is, under f=30 criteria, the correct identification rate is still greater than 80%, under the worse condition $P_{c}=0.6$, if x is carefully chosen, as shown in the gray region in Table 4. However, once the single correct rate $P_{c}$ is less than 0.6, the total identification rate cannot be improved by time series analyzing no matter what the variables are. Therefore, it can be viewed as a good threshold for the features selection used to distinguish the real/fake face.

**Figure 16:**
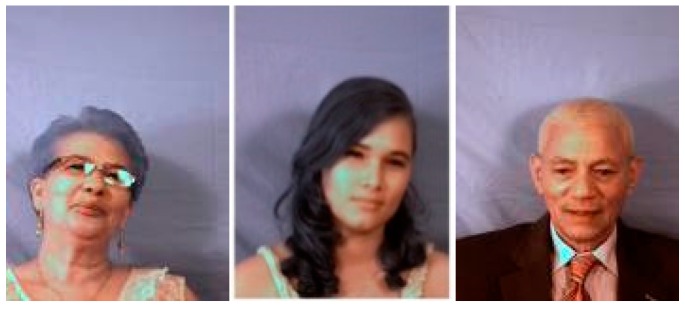
Samples of face with high reflected regions.

The second process to improve the detection rate is to reduce the influence from the high reflected regions in the face. As shown in Figure 16, it is observed that detection of faces with strong reflected regions by the environment light has greater detection error. Thus, it is intuitive to detect and remove such regions to improve the detection rate. It is also observed from the experimental results that the FRR can be reduced significantly to 0.016 for a single shot image. That is, most live faces, which are determined as fake, can be detected correctly.

Finally, detecting the different parts of a face separately and then determining whether it is fake, instead of determining authenticity by the global face detection, can reduce the error probability. This is because the face detection error due to influenced regions can be omitted. To perform this, six regions (eyes, nose, eyebrows, and mouth) are segmented and trained for detection. If the positive detection number is greater than 4, than the face is determined as a live face; otherwise it will be thought of as a fake face. The simulation results also show that the average detection rate can be improved to 0.968 for a single shot image.

To sum up, compared with some present non-intrusive anti-spoofing methods in the reviewed paper [24], the proposed method has either better or comparable spoofing detection accuracy for still/moving images by gathering a series of face samples, while the computational complexity and the system cost are kept low enough. Hence, this method is much more suitable for implementation in a handheld device.

## 5. Conclusions and the Future Works

In this paper, a fast and effective system which is composed of optical image sensor and expert decision-making core for spoofing face detection has been proposed and verified to improve the reliability of a face authentication system. Via analyzing the specific features of the displayed fake face reproduced by the high definition display monitor, it is possible to effectively verify the dynamic authentic images and the spoofed images (or videos) by analyzing the relations between the chrominance characteristics and the saturation of the captured face images.

The experimental results show that not only is the correct identification rate high enough, but the total reliability of the identification system can be made trustworthy by simply adjusting the analyzation period variables, the number of the photos captured by the camera as well as those of the photos determined to be authentic. That is, the study result has achieved outstanding success results, greater than 99% success rate, in terms of face spoofing detection.

However, to simplify the experiment implementation, the optimized network architecture has not been designed for this study. It is believed that the accuracy of the detection can be effectively improved if a more appropriate network mode is adopted in the future, Moreover, feature vectors, determined by the face component, which is fed into the input layer of PNN can be modified to increase the average correct identification rate for a single frame and thus to increase the performance of the spoofing detection method.

## Figures and Tables

**Table 1:** The control variables description for training vectors.

Independent Variables	Glasses: if the subject wears glasses
	Saturation of the left eye: the average saturation of the left eye’s image
	Chrominance of the left eye: the average chrominance of the left eye’s image (within the eye’s region)
	Saturation of the right eye: the average saturation of the right eye’s image
	Chrominance of the right eye: the average chrominance of the right eye’s image (within the eye’s region)
	Saturation of the left eyebrows: the average saturation of the left eyebrows’ image
	Chrominance of the left eyebrows: the average chrominance of the left eyebrows’ image (within the eyebrows’ region)
	Saturation of the right eyebrows: the average saturation of the right eyebrows’ image
	Chrominance of the right eyebrows: the average chrominance of the right eyebrows’ image (within the eyebrows’ region)
	Saturation of the nose: the average saturation of the nose image (within the region of the nose)
	Chrominance of the nose: the average chrominance of the nose image (within the region of the nose)
	Saturation of the mouth: the average saturation of the mouth image (within the region of the mouth)
	Chrominance of the mouth: the average chrominance of the mouth image (within the region of the mouth)
Dependent Variable	Determined Result: if the image is authentic

**Table 2:** Experimental results for spoofing face detection under a single shot condition.

$ER_{ave}=\frac{No.\;\mathrm{error}\;\mathrm{detection}\;}{No.\;\mathrm{test}\;\mathrm{images}\;}=\frac{1061}{4557}=0.233$	
***FAR*** (single shot)	$FAR=\frac{No.\;\mathrm{error}\;\mathrm{detection}\;}{No.\;\mathrm{fake}\;\mathrm{faces}\;}=\frac{433}{2780}=0.156$
***FRR*** (single shot)	$FRR=\frac{No.\;\mathrm{error}\;\mathrm{detection}\;}{No.\;\mathrm{true}\;\mathrm{faces}\;}=\frac{628}{1777}=0.353$
ER≐FAR=0.156	A life face is easy to be confirmed by the previous algorithms, thus ***FRR*** can be ignored

**Table 3:** Some practical variables and the corresponding results.

f	x	$P_{T}$
10	8	83.05%
10	7	95.42%
10	6	99.12%
15	12	83.40%
15	11	94.41%
15	10	98.52%
15	9	99.69%
15	8	99.95%

**Table 4:** Identification results for various $P_{c}$ under f=30.

$P_{c}$	x	$P_{T}$
0.6	16	82.46%
0.6	18	57.85%
0.7	16	98.31%
0.7	18	91.55%
0.8	16	99.98%
0.8	18	99.69%
